# Decrease in retinal neuronal cells in streptozotocin-induced diabetic mice

**Published:** 2012-06-01

**Authors:** Yang Yang, Danna Mao, Xinke Chen, Ling Zhao, Qing Tian, Chenggang Liu, Bo Lei Shanbi Zhou

**Affiliations:** Department of Ophthalmology, the First Affiliated Hospital of Chongqing Medical University, Chongqing Key Laboratory of Ophthalmology, Chongqing Eye Institute, Chongqing, China

## Abstract

**Purpose:**

Little is known about retinal neuronal loss in the retinas of diabetic mice. The purpose of this study was the quantitative assessment of retinal neural cell number in diabetic mice.

**Methods:**

Five-week-old C57BL/6 mice were used as a diabetic model with streptozotocin. Mice were studied over the course of 6 and 12 weeks after the onset of diabetes. Intraocular pressure (IOP) was measured with a noninvasive TonoLab tonometer. The retinal ganglion cells (RGCs) were counted at two different time points after the induction of diabetes and examined using the immunofluorescence technique and quantitative analysis.

**Results:**

The diabetic mice had significantly elevated IOP levels at 6 and 12 weeks after the onset of diabetes compared with the age-matched control mice (p<0.01 and p<0.001, respectively). The temporal course of Brn3a+ RGC and Neuronal Nuclei+RGC (NeuN+ RGC) loss induced by intraperitoneal injection of streptozotocin followed a similar trend. At 6 and 12 weeks after the onset of diabetes, the number of Brn3a+ RGCs (p<0.05 at 6 weeks; p<0.001 at 12 weeks) and NeuN+ RGCs (p<0.05 at 6 weeks; p<0.001 at 12 weeks) was significantly lower in diabetic mice than age-matched control mice. In the retinal flatmounts, the number of Brn3a+ RGCs (p<0.05 at 6 weeks, p<0.01 at 12 weeks) was also significantly lower in diabetic mice than control mice. The IOP in diabetic mice was negatively related with RGCs in cross sections. The cut-off value of IOP was 14.2 mmHg for diabetes.

**Conclusions:**

This is a specific quantitative study of neural cell loss in the retina during diabetes. These data suggest that retinal neural cell reduction occurs in diabetic mice. It indicates that RGC loss may be an important component of diabetic retinopathy.

## Introduction

Diabetic retinopathy (DR), the leading cause of blindness in working-age adults, has enormous public health implications: It has been predicted that the number of people at risk of developing vision loss from diabetes worldwide will double over the next 25 years [1]. DR has long been recognized as a vascular disease that develops in most patients, and it is believed that the visual dysfunction that develops in some diabetics is due to the vascular lesions used to characterize the disease [2,3]. Little is known about the pathological changes in retinal neurons that are likely to occur in DR. However, DR has also recently been identified as a neurodegenerative disease of the retina. Much evidence suggests that changes in the functional molecules and viability of neurons in the retina occur early after the onset of diabetes, preceding the vascular complications in humans and experimental animals [4]. Recent studies have emphasized the importance of diabetes-induced neuronal damage in the retina at an early stage of disease progression [5-9]. The loss of cells due to diabetes is likely to include retinal ganglion cells (RGCs) and other neurons [10]. However, glaucoma is often associated with high intraocular pressure (IOP) as a result of RGC death [11,12]. Diabetes has also been associated with elevated IOP [13]. Therefore, in this study, we have measured the IOP in diabetic and age-matched control mice.

The neural retina is composed of diverse neurons characterized by morphological and biochemical criteria, as well as numerous neural networks formed through chemical and electrical synapses between these neuronal processes. These neural components are arranged into retinal layers. RGCs, located in the ganglion cell layer (GCL), because of their high sensitivity to cellular damage and neurotoxicity, offer a unique and effective model to study the mechanism of neurodegenerative disease progression [14-20]. There are two reliable and efficient neuron-specific proteins (neuronal nuclei [NeuN] [21] and Brn3a [22]), which are used as markers to identify and quantify RGCs in normal and optic nerve–injured retinas. Thus, in this study, we have quantitatively analyzed RGCs that are labeled using Brn3a and NeuN, examining the diabetes-induced loss of RGCs in the retinas.

At present, the most widely accepted animal model for the evaluation of retinal complication in diabetes is that of streptozotocin (STZ)-induced diabetic rats. The retinal lesions observed in the diabetic rats resemble the initial process of DR that occurs in humans [23]. It has also been shown that in using STZ-treated rats, significantly more neuronal cells undergo loss in the retinas of diabetic rats than in control animals [10,24]. Mice have been used less frequently as a model in studies of DR. However, the mouse has been extensively studied in many other experimental fields because of the availability of transgenic mice, their similar genetic background, relatively quick breeding, and low cost. Moreover, it is apparent that this species exhibits features of DR. Previous studies have demonstrated that retinal neuronal cell loss occurs in diabetic mice [25]. It is thought that diabetic mice may be appropriate and valuable models for studies of neuronal cells loss involved in diabetes. However, reports on the effects of diabetes on retinal neurons in mice have also been contradictory. An increased frequency of decrease retinal neurons has been reported in some [25-27], but not all [7,24], studies of diabetic mice. To clarify these contradictions and provide detailed data on retinal neuronal cell loss after the induction of diabetes, we investigated the occurrence and time course of diabetes-induced changes in RGCs in the retina of C57BL/6 mice, a commonly used mouse strain.

## Methods

### Induction of diabetes in mice

Male C57BL/6 mice were obtained from the animal center at Chongqing Medical University (Chongqing, China), and five-week-old mice were used. All animal procedures performed in this study complied with the ARVO statement for the use of Animals in Ophthalmic and Vision Research. Mice received an intraperitoneal injection of 60 mg/kg STZ (Sigma-Aldrich, St. Louis, MO) dissolved in sodium citrate buffer (0.01M, pH 4.5) on three successive days. After 1, 6, and 12 weeks of injection with STZ, blood glucose was measured using a glucometer. Fasting blood glucose levels higher than 250 mg/dl were considered to be diabetic. Age-matched, nondiabetic C57BL/6 mice were used as controls.

### Intraocular pressure measurement

IOP was measured using the TonoLab rebound tonometer for rodents (Colonial Medical Supply, Franconia, NH) according to the manufacturer’s recommended procedures. The distance would be 1–4 mm from the tip the probe to the cornea of the eye. Measure takes place by lightly pressing the measurement button. The tip of the probe had hit the central cornea Awake adult C57BL/6 mice were restrained for applanation tonometry in a polyethylene cone (Decapicone; Braintree Scientific, Inc., Braintree, MA) with its apex trimmed to allow exposure of the head [28]. After a few minutes of acclimation, the mice usually appeared calm and comfortable. IOP was measured noninvasively in C57BL/6 diabetic mice and age-matched, nondiabetic C57BL/6 mice at 6 and 12 weeks after the onset of diabetes.

### Retinal section and flatmount preparation

All animals were euthanatized by an overdose of carbon dioxide asphyxiation followed by cervical dislocation. For studies using frozen sections, eyes were enucleated, embedded in optimum cutting temperature (OCT) and flash frozen. Then, 10 μm thick sections, which included a full length of retina approximately along the horizontal meridian, passing through the ora serrata and the optic nerve in both the temporal and nasal hemispheres, were cut and mounted on slides. In the flatmounts, the eyes were immediately enucleated and fixed with 4% paraformaldehyde at 4 °C for 4 h, and the retinas were dissected from the ora serrata. Retinal flatmounts were prepared by making six radial incisions and then carefully placing the retinas on anti-off slides (DingGuo, Beijing, China).

### Immunohistofluorescence

#### Radial sections

The sections were stained with a combination of goat anti-Brn3a (C-20) antibody (Santa Cruz Biotechnology, Heidelberg, Germany) and anti-NeuN (A60) antibody (mAb; Chemicon, Temecula, CA). Goat anti-Brn3a (C-20) and anti-NeuN (A60) antibody diluted 1:200 in phosphate-buffered saline (PBS, containing: NaCl 137 mmol/l, KCl 2.7 mmol/l, Na_2_HPO 10mmol/l, KH_2_PO_4_ 2 mmol/l) with 0.1% Triton-100. The mixtures were blocked in 5% donkey serum in PBS with 0.1% Triton-100 and incubated overnight at 4 °C. The goat anti-Brn3a (C-20) antibody was detected with Alexa Fluor-568 donkey antigoat IgG (H^+^L) antibody (Invitrogen–Molecular Probes, Barcelona, Spain). The NeuN (A60) antibody was detected with a donkey antimouse Cy3 antibody (Jackson ImmunoResearch, West Grove, PA). The secondary antibodies were incubated for 45 min at room temperature. The sections were counterstained with 4’, 6’-diamino-2-phenylindole (DAPI) and a fluorescence microscope (DMIRB; Leica, Bannockburn, IL) was used to capture the images.

#### Flatmounted retinas

Retinas from both eyes were dissected as flattened wholemounts, as previously reported [29]. Retinas from eyes were dissected as flattened whole-mounts by making four radial cuts (the deepest one in the dorsal pole), post-fixed for an additional hour in the same fixative, rinsed in 0.1 M PBS, mounted vitreal side up on subbed slides and covered with anti-fading mounting media containing 50% glycerol and 0.04% p-phenylenediamine in 0.1 M sodium carbonate buffer (pH 9.0). The retinas were permeabilized in PBS 0.5% Triton X-100 for 15 min at −80 °C, rinsed in new PBS 0.5% Triton X-100, and incubated overnight at 4 °C with goat anti-Brn3a antibody diluted 1:100 in blocking buffer (PBS, 2% BSA, 2% Triton X-100). Then, the retinas were washed three times in PBS and incubated for 2 h at room temperature with the secondary antibody diluted 1:500 in blocking buffer. Finally, they were thoroughly washed in PBS and mounted vitreous side up on subbed slides and covered with glycerin solution.

### Image analysis and retinal ganglion cell counts

Images were collected with a CCD camera (Retiga Exi; Qimaging, Burnaby, BC, Canada). Six visual fields were sampled from the posterior portion of each flatmounted retina (each visual field representing a 13.3 μm length and 10 μm width of the retina) under fluorescence microscopy; fluorescently labeled ganglion cells were counted in six regions in the six quadrants of each retina at approximately the same distance (1 mm) from the edge of the optic disc. In radial sections, we chose the first image from the optic nerve attachment to the retinal periphery to count the number of RGCs. The location of the field was specified to avoid variations in RGC density as a function of distance from the optic disc. The average number of RGCs per field was calculated in each retina.

### Statistical analysis

Statistical analyses were undertaken using SPSS (version 13.0; SPSS Inc., Chicago, IL). Results are presented as mean±standard deviation (SD). The Mann–Whitney test or Student *t* test was used to compare two groups of results, and one-way ANOVA followed by Bonferroni’s multiple comparison was used to compare among three or more groups. The correlation between IOP and RGCs was examined by Pearson’s correlation analysis. Receiver operating characteristic curve (ROC) curve analysis was performed by Medcalc v 7.4.1.0. Differences were regarded as significant when p<0.05.

## Results

### Bodyweight and blood glucose of diabetic and age-matched control mice

Table 1 shows the bodyweight and blood glucose levels of diabetic and age-matched control mice. Diabetic mice showed a significant decrease in bodyweight and a significant increase in blood glucose compared with aged-matched controls. At 1 week after the onset of diabetes, bodyweight and blood glucose levels were measured in control and diabetic mice (17.03±0.84 g versus 15.90±0.48 g, p=0.002; 148.2±21.7 mg/dl versus 285.3±25.4 mg/dl, p<0.001). Compared to the control group, the blood glucose levels of diabetic mice increased markedly from 6 to 12 weeks (153.2±16.3 mg/dl versus 493.0±14.6 mg/dl, p<0.001; 155.9±26.3 mg/dl versus 609.2±12.9 mg/dl, p<0.001). However, in contrast to control groups, the bodyweight of diabetic mice decreased significantly at the same time points (23.6±0.71 g versus 15.72±0.69 g, p<0.001; 25.83±1.02 g versus 14.30±0.55 g, p<0.001).

**Table 1:** Effects of STZ-Induced diabetes on bodyweight and blood glucose levels in different groups after 1, 6, 12 weeks (n=20/group)

	**Bodyweight (g)**		**Blood glucose (mg/dl)**	
**Time**	**Control**	**T1DM**	**Control**	**T1DM**
1 week	17.03±0.84	15.90±0.48**	148.2±21.7	285.3±25.4***
6 weeks	23.62±0.71	15.72±0.69***	153.2±16.3	493.0±14.6***
12 weeks	25.83±1.02	14.30±0.55***	155.9±26.3	609.2±12.9***

Data are expressed as the mean±SD. The significantly different from age-matched control mice (** indicates p<0.01, *** indicates p<0.001).

### Intraocular pressure in diabetic and age-matched control mice

**Figure 1:**
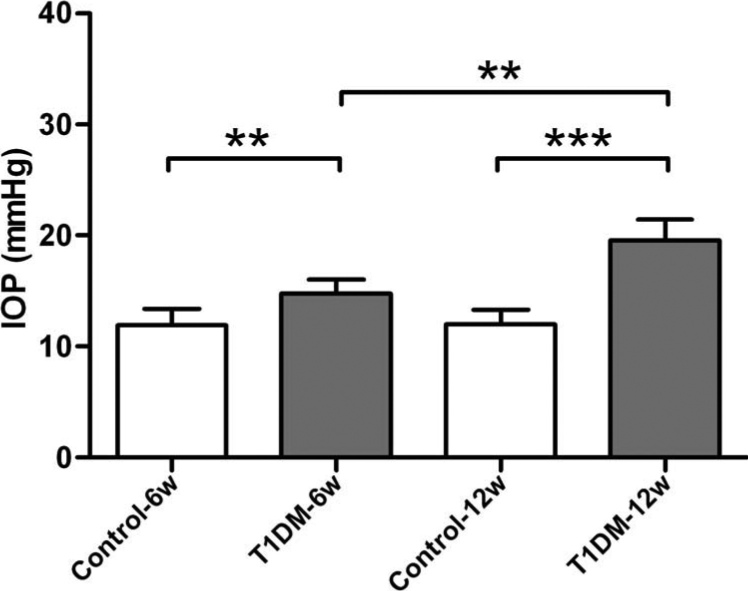
Intraocular pressure in streptozotocin-induced diabetic and control mice. The 6- and 12-week diabetic mice had a significantly higher intraocular pressure (IOP) than the age-matched control mice. Compared with 6-week diabetic mice, 12-week diabetic mice also had significantly increased IOP (n=8/group, ** indicates p<0.01, *** indicates p<0.001).

Loss of RGCs occurs in many ophthalmic conditions such as glaucoma [20] and diabetes [30]. Some of the molecular and cellular mechanisms that may be involved in RGC loss in ocular conditions are associated with elevated IOP. Therefore, in this study, we have compared the IOP of diabetic mice with that of age-matched controls (Figure 1). The levels of IOP in 6- and 12-week diabetic mice was significantly higher than those of age-matched control mice (11.94±1.53 mmHg versus 14.78±1.20 mmHg, p<0.01; 12.02±1.33 mmHg versus 19.52±1.89 mmHg, p<0.001). On the other hand, there was also a significantly elevated IOP comparing 6-week diabetic mice with 12-week diabetic mice (14.78±1.20 mmHg versus 19.52±1.89 mmHg, p<0.01).

### Neural cell loss in the diabetic retina section

**Figure 2:**
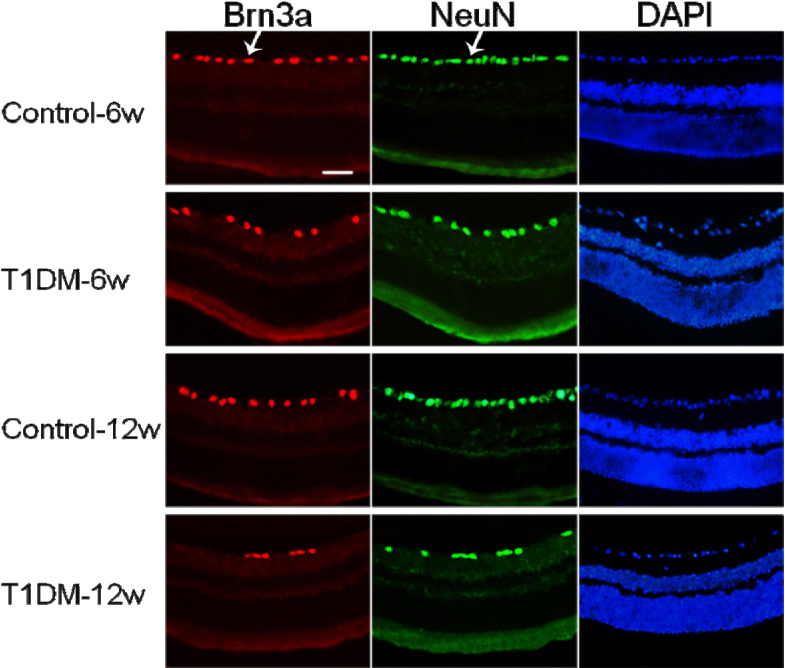
The retinal ganglion cells were double labeled with Brn3a and NeuN in the same radial sections. Left: Brn3a signal (red); middle: NeuN signal (green); right: DAPI (blue). Arrows representative Brn3a-labeled retinal ganglion cells (RGCs) (red) and NeuN-labeled RGCs (green). All images were obtained at 40× magnification. Bar: 2 μm.

**Figure 3:**
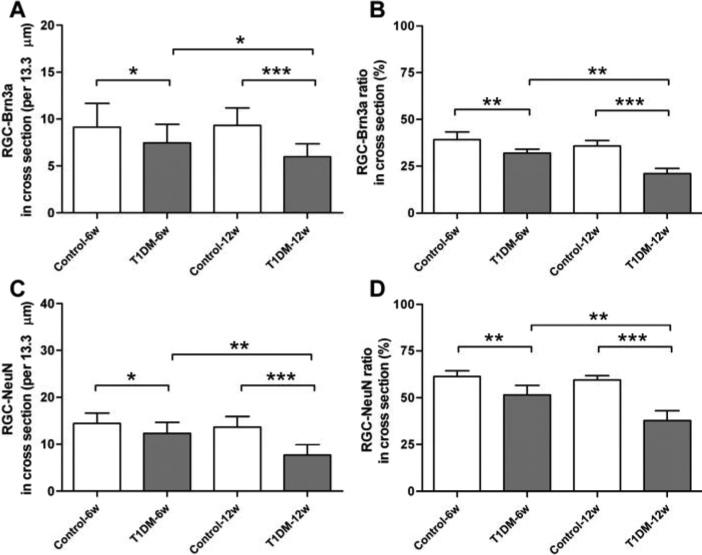
Quantification of retinal ganglion cell loss in the ganglion cell layer. Cell loss was assessed by counting the cells with Brn3a (**A**) and NeuN (**C**) immunoreactivity. The number of retinal ganglion cells (RGCs) exhibits a significantly difference in diabetic and control mice (n=10/group). In the percentage of RGCs for Brn3a (**B**) and NeuN (**D**), both markers were calculated in relation to the number of cells counted. Data are expressed as means±standard deviation (SD; * indicates p<0.05, ** indicates p<0.01, *** indicates p<0.001).

Neurodegeneration of the retina is a critical component of DR and a significant loss of RGCs has been reported in STZ-induced diabetic mice [25,26]. We quantitatively analyzed RGCs in radial sections of the retina in diabetic mice using both the Brn3a and NeuN markers (Figure 2). Compared to control mice, the numbers of Brn3a^+^ RGCs (7.46±1.75 versus 8.93±2.14 cells/13.3 um at 6 weeks, p<0.05 and 5.96±1.37 versus 9.04±1.84 cells/13.3 um at 12 weeks, p<0.001) was significantly lower in diabetic mice (Figure 3A). Thus, the number of Brn3a^+^ RGCs exhibited a 7% loss over 6 weeks and a 15% loss over 12 weeks (Figure 3B). In contrast to the control mice, the numbers of NeuN^+^ RGCs (14.43±2.41 versus 12.3±2.39 cells/13.3 um at 6 weeks p<0.05 and 13.65 ± 2.22 versus 7.75±2.15 cells/13.3 um at 12 weeks, p<0.001) was also significantly decreased in diabetic mice (Figure 3C). Thus, the number of NeuN^+^ RGCs showed a 10% loss over 6 weeks and 22% loss over 12 weeks (Figure 3D).

### Quantification of the retinal flatmounts of Brn3a^+^ retinal ganglion cells

**Figure 4:**
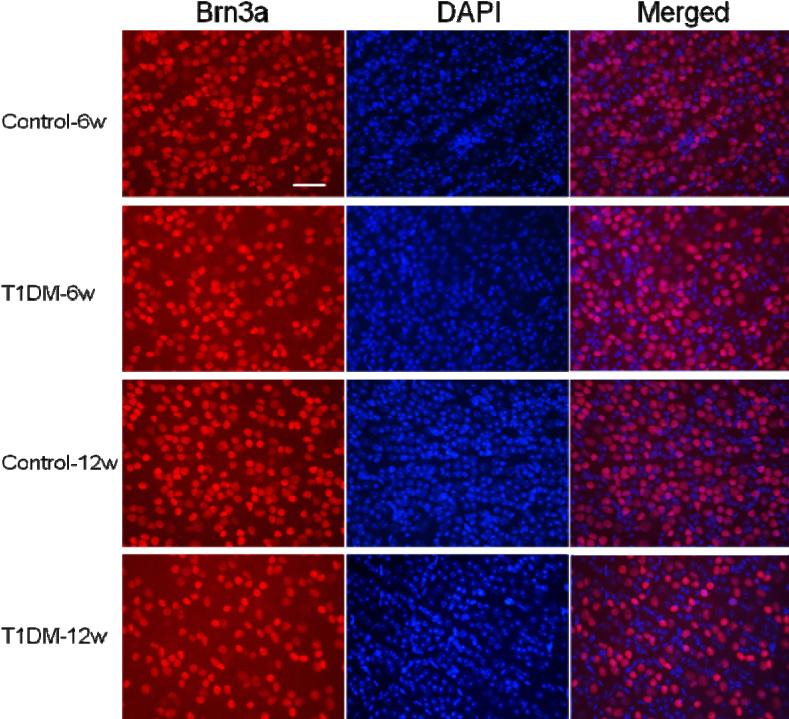
Brn3a-labeled retinal ganglion cells in flatmount retinas. Left: Brn3a signal (red); middle: 4’,6’-diamino-2-phenylindole (DAPI) signal (blue); right: superimposition of both images. The density of Brn3a+ RGCs in 6 and 12 weeks’ onset of diabetes and control mice are shown in the images (each visual field represents a 133 μm^2^ area). All images were obtained at 40× magnification. Bar: 2 μm.

**Figure 5:**
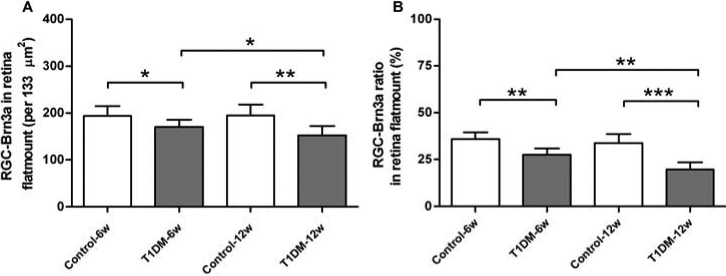
Quantification of Brn3a-labeled retinal ganglion cells in retinal flatmounts. **A**: Compared to control groups, diabetic mice have significantly decreased numbers of retinal ganglion cells (RGCs; n=10/group). **B**: There is a similar result of percentage of RGCs labeled with Brn3a. The percentage of RGCs for marker combination was calculated in relation to the number of RGCs. Data are expressed as means±standard deviation (SD; * indicates p<0.05, ** indicates p<0.01, *** indicates p<0.001).

To further demonstrate the diabetes-induced neuronal loss, we quantitatively analyzed Brn3a^+^ RGCs by a manual counting process in whole flatmounted retinas (Figure 4). We found that the numbers of Brn3a^+^ RGCs (170±15.34 versus 193.5±21.38 cells/133 um^2^ at 6 weeks, p<0.05; 152.2±20.10 versus 194.5±23.29 cells/133 μm^2^ at 12 weeks, p<0.01) of the diabetic mice were significantly lower than those of the age-matched control mice. Thus, there was an 8% loss over 6 weeks and 14% loss over 12 weeks (Figure 5). The number of Brn3a^+^ RGCs also showed a significant decrease (p<0.05) at 6-week diabetic mice compared with 12-week diabetic mice.

### Correlation between intraocular pressure elevation and retinal ganglion cell loss in diabetic mice

**Figure 6:**
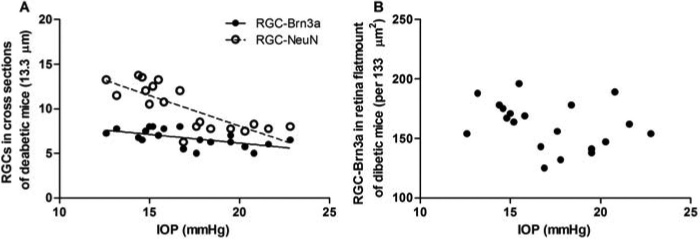
The correlation between intraocular pressure and retinal ganglion cells in diabetic mice. **A**: Retinal ganglion cells (RGCs) were negatively related with intraocular pressure (IOP) in cross sections (RGC-Brn3a: r=-0.589, p<0.01; RGCs-NeuN: r=-0.789, p<0.001, respectively, n=20). **B**: There was no correlation between RGCs and IOP in wholemount retinas.

In diabetic mice, we found increased IOP and decreased RGCs. To further investigate the relation between these results, we analyzed the correlation between IOP and RGCs. IOP was negatively correlated with RGCs in cross section (RGCs-Brn3a: r=-0.589, p<0.01; RGCs-NeuN: r=-0.789, p<0.001; Figure 6A), and was not correlated with RGCs-Brn3a in wholemount retinas (Figure 6B).

### ROC curve of intraocular pressure

**Figure 7:**
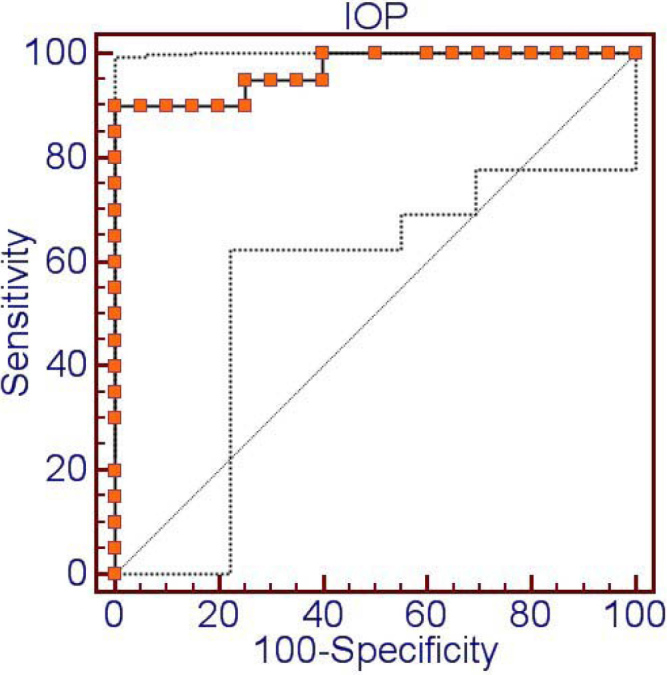
The ROC curve of intraocular pressure for diabetes. The estimated cut-off value of intraocular pressure (IOP) was 14.2 mmHg, with 90% sensitivity and 100% specificity for the diabetes (area under the curve=0.967, p<0.0001).

ROC curve analysis was performed to establish a threshold IOP for the diabetes. The estimated cut-off value of IOP was 14.2 mmHg, with 90% sensitivity and 100% specificity for the diabetes (area under the curve=0.967, p<0.0001; Figure 7).

## Discussion

In the current study, we examined the loss of neuronal cells in the retinas of diabetic mice. Several important findings emerged from the present study. First, diabetic C57BL/6 mice exhibited progressive loss of RGCs in the GCL during diabetes. By six weeks after induced of diabetes, the number of RGCs was reduced by approximately 9% of the normal number. However, 12 weeks after induction of diabetes, the number of RGCs exhibited a 19% loss compared to normal. Therefore, a gradual decrease in the number of RGCs was demonstrated. There is general agreement that all rat strains have shown RGC loss or damage in diabetes [10,21,31-38]. However, the small size of the mouse eye presents unique challenges in determining cell loss in the mouse retina. The mouse is a useful model to investigate ocular diseases because of the availability of transgenic mice, their similar genetic background, relatively quick breeding, and low cost. Moreover, mouse and human eyes have similar anatomic structure, including a well—defined trabecular meshwork, Schlemm’s canal, ciliary body, and vascularized retina, outflow pathways, and response to IOP-lowering drugs. Although mouse models involve special problems, the ability to use genetically modified mice to study the pathogenesis of DR provides a major advantage over the use of any other animal species, offsetting the potential hardships. In this study, we counted surviving RGCs in retinal sections using both the Brn3a and NeuN markers. However, the average numbers of RGCs in the both groups showed an apparent difference. Two factors may explain this. First, Brn3a (C-20), a nuclear protein, is an endogenous marker of RGCs, whereas a NeuN clone exists (A60), and this reacts with an uncharacterized nuclear protein. The neural retina is composed of diverse neurons, and these neural components are arranged into retinal layers. This increase in conventional synapses may represent increased amacrine synaptogenesis onto RGC dendrites. However, the amacrine cells can make up over 50% of the cells in the GCL of retina. Moreover, NeuN may also label the amacrine cells in the GCL [39]. Thus, NeuN expression reflects not only ganglion cells but also displaced amacrine cells, resulting in the difference between RGCs labeled with NeuN and Brn3a. Second, Brn3a is an affinity purified polyclonal antibody and NeuN is a monoclonal antibody. At present, our results and those of others [25] have indicated that loss of RGCs occurs in the retina of C57BL/6 mice in association with the time course of DR, similar to findings in diabetic rats and humans. However, there has been a controversy over whether or not diabetes causes RGC loss in this mouse strain. One group of investigators reported that STZ-induced diabetes in this strain rapidly resulted in extensive loss of RGCs, with 20%–25% fewer cells in the GCL compared with age-matched control mice after only 14 weeks of diabetes [25]. In contrast, other investigators using this same strain found no evidence of RGC degeneration even after one year of diabetes, based on counts of the number of cell bodies in the RGC layer of retinal cross sections [7,24]. Here, we reported that neural cell loss increases after 6 weeks of diabetes and remains further elevated during a 12-week period. These data confirm that retinal cell loss is not specific to STZ diabetic rats. In a separate time-course study, we found 9% and 19% decreases in the number of RGCs, respectively, at 6 and 12 weeks after induction of diabetes. Similarly rapid decreases in RGC counts in diabetic animals have been reported by other researchers using different methods to assess the number of RGCs. For example, Barber et al. [40] observed a 23.4% loss of cells in the retinal ganglion layer at 22 weeks after the induction of diabetes. Thus, results from other laboratories confirm our study’s result of a rapid loss in RGCs.

The second important finding is that IOP was elevated in diabetic mice and the cut-off value of IOP for diabetes was 14.2 mmHg. IOP was negatively correlated with RGC loss, suggesting that elevated IOP may be associated with RGC loss in diabetic mice. Although the mechanism that underlies RGC damage in this disease is poorly understood, previous studies have suggested that elevated IOP might play a key role in RGC loss or damage [11,12,41]. The hydrostatic pressure of the aqueous fluid compartment of the eye is defined as IOP. Maintenance of IOP within a narrow range is essential for the normal health of the eye. The IOP is kept within these limits by the production rate of aqueous humor into the posterior chamber of the eye and its drainage out of the anterior chamber angle. Alterations in aqueous flow have been described in humans with diabetes, and it is thought that this contributes to diabetes-related changes in the eye [42,43]. It was reported that aqueous flow decreased by 15% in patients with type 1 diabetes without evidence of microvascular complications compared to healthy control participants [42]. Insulin and glucose levels are known to have an effect on blood flow in tissue [44,45], and blood flow is a determinant of aqueous flow [46]. This agrees with several recent epidemiologic studies that report an association between diabetes and elevated IOP [47-49]. It has been reported recently that diabetic patients have lower corneal hysteresis than healthy controls, which could explain, in part, the higher IOP in this group [50,51]. In animal studies, Kanamori and coworkers [52] showed that chronic ocular hypertension (four weeks, from 13 to 29 mmHg), induced by cauterizing three episcleral veins, was more detrimental in STZ-diabetic rat retinas compared with control eyes. The authors showed that there was an increased loss in the inner retina in high IOP–challenged STZ-treated rats. This suggests that existing RGC dysfunction or loss in diabetes is produced by IOP stress and allows the potential mechanisms linking diabetes and glaucoma to be considered. The role of IOP in diabetes will continue to be investigated, as further research in these areas would be of interest.

At present, there is still no consensus on the best pharmacological target for DR, but the number of RGCs lost has clearly been shown to participate in diabetes [7,10,25,30,37,40,53-56]. However, the molecular mechanism by which the cells decrease or develop other structural abnormalities is not yet clear. Common theories suggest that inflammation, oxidative stress, or exposure to advanced glycation end products might contribute to retinal pathologies, including RGC loss. Currently, there have been too few studies to clearly indicate the biochemical mechanisms leading to RGC loss in diabetes, but this research has provided important information to initiate further work. RGCs serve as an ideal model to evaluate the neuroprotective effects of steroid hormones, and at the same time, can offer important insights for the development of novel treatments for retinal degeneration and other neurodegenerative diseases. It is important to recognize that most of these studies have also focused on preventing RGC degeneration, but the effects of these and other therapies on dendritic field morphology and other features important for RGC function have not been assessed. The focus on RGCs in diabetes should not be restricted to degeneration. Information about diabetes-induced dysfunction, anatomy, and response properties is expected to provide considerable insight into the visual dysfunction that develops in some diabetic patients. This could further aid in the prevention of early neuronal degeneration in ongoing diabetic retinopathy.
